# Comparison of Silk Glands of Diapause and Non-Diapause Larval *Sitodiplosis mosellana*

**DOI:** 10.1673/031.012.8101

**Published:** 2012-07-12

**Authors:** Yiping Li, Junxiang Wu, Weining Cheng, Weiwu Song, Xiangqun Yuan

**Affiliations:** Key Laboratory of Applied Entomology, College of Plant Protection, Northwest A & F University, Yangling, Shaanxi 712100, China

**Keywords:** orange wheat blossom midge, salivary gland, ultrastructure

## Abstract

The wheat midge, *Sitodiplosis mosellana* (Géhin) (Diptera: Cecidomyiidae), is one of the most serious pests of wheat worldwide. It overwinters as cocooned larvae in diapause and non-diapause forms. The cocoon is made of silk from the salivary glands. The silk glands, therefore, play an important role in the *S. mosellana* diapause. In the present study, the ultra-structures of the silk glands between diapause and non-diapause larvae were examined by electro and light-microscopically. The silk glands consist of 156 cells organized like moniliform particles. Although silk gland cells of both diapause and non-diapause larvae contain developed organelles, including the endoplasmic reticulum, dictyosome, mitochondria, and lipid droplet, the organelles in non-diapause larvae are more developed than those in diapause larvae. These morphological characteristics of the silk glands in the diapause and non-diapause larvae can be used to distinguish the diapause status of the larvae.

## Introduction

Wheat midges, *Sitodiplosis mosellana* (Géhin) (Diptera: Cecidomyiidae), are found in most parts of the world where wheat is grown (Yuan et al. 2003; Doane and Olfert 2008). Two disastrous outbreaks of *S. mosellana* over large areas of China were recorded in the 1950's and the 1980's (Yuan 2004). Reduction in wheat production due to this insect pest is usually 10–20%, but severe outbreaks can destroy the crop completely (Yuan et al. 2003). In recent years, *S. mosellana* has been effectively controlled, but it still remains a serious pest in some regions of China, and has the potential to spread (Yuan 2004, Cheng et al. 2009a). The diapause and diapause polymorphism in *S. mosellana* are some of the important factors that govern serious outbreaks of this pest insect (Wise and Lamb 2004, Wu et al. 2004a). *S. mosellana* is known to have one generation per year. It overwinters, and spends the summer as a diapausing cocooned larva, or an exposed larva in soil. The pupation, eclosion, and damage to the crop usually start in the spring, when wheat is approaching its shooting stage (Yuan et al. 2003; Doane and Olfert 2008), although the diapause can be prolonged if overwintering larvae encounter adverse conditions during pupation and eclosion in the spring. The longest diapause recorded lasted nearly 12 years (Hu and Zhang 1995).

The silk glands of insects are responsible for the secretion of the silk used for shelter or cocoon formation (Sehnal and Akai 1990, Victoriano 2007). In cocoon-making insects, the silk glands, which secrete a protein or viscid liquid that hardens into silk upon exposure to air, play a vital role in protecting the insect from adverse environmental conditions (Sasaki and Tashiro 1976, Akai 1984, Silva-Zacarin et al. 2003). The research on understanding the diapauses of *S. mosellana* has made significant progress in recent years, especially on hormoneregulation (Cheng et al. 2009b), changes in such constituents as saccharides (Wu et al. 2004b), lipids (Wu and Yuan 2004), proteins (Cheng et al. 2009a), metabolic enzymes (Cheng et al. 2009c), and molecular mechanisms (Wang et al. 2007).

Morphological characteristics are useful for practical work such as identification of species, and studying ecotype, because they can be observed directly. For example Hu (1988), in his initial observations on the external morphology of the silk gland in *S. mosellana5* found that the silk glands in both non-diapause and cocooned larvae were comprised of a pair of long, hyaline, sack-like structures. The ultrastructure of the silk glands, however, was not previously described. Therefore, the objectives of our study were to determine the ultrastructure of the silk glands in diapause and non-diapause larvae of *S. mosellana* by using a dissecting microscope and an electron microscope, to distinguish the diapause larval status based on the morphological characteristics, and to determine the relationship between the ultrastructure of the silk glands and diapause. The results will provide vital information for the development of strategies on how to manage this important insect pest.

## Materials and Methods

### The tested insects

*S. mosellana* infested ears of wheat were collected from Xushui County, Hebei Province, China, in May 2004, when wheat was at the yellow or mealy ripening stage. Non-diapause larvae were collected directly from infested wheat ears. To obtain diapause larvae, the wheat ears were placed in a field at Northwest A&F University, Yangling, Shaanxi, China. The larvae were dropped on the ground, and they entered into the soil to overwinter. These diapaused larvae were collected from the soil in mid-December as described by Wu et al. (2005). The collected diapaused and non-diapaused larvae were fixed in 3% glutaraldehyde before they were used.

### Materials and equipment

The following reagents were obtained from Sigma Corporation: 5% methylene blue trihydrate, 3% glutaraldehyde, phosphate buffer, 25% glutaraldehyde, osmic acid, acetone, Epon 812 epoxide resin, dodecenyl succinic anhydride (DDSA), methyl nadic anhydride (MNA), uranyl acetate, plumbi nitras, citrate sodium, and tri (dimethylaminomethyl) phenol (DMP-30).

A compound microscope (Eclipse 80i; Nikon, Tokyo, Japan), a dissecting microscope 94 (SMZ 660; Nikon, Japan), and an electronic microscope (JEM-2000EX; JEOL Co., 95 Tokyo, Japan) were used in this study.

### The observation method

Observations under the light microscope. The larvae were placed on a waxed dish under the light microscope by using two dissecting needles, one to press the head at a point close to the mouthparts, and the other to press the caudal end. The mouthparts were then removed from the body gently and quickly, so that the internal organs flowed out from the cut end, exposing two halves of the sialisterium. A drop of physiological saline was put on a glass slide, and the sialisterium was transferred to the physiological saline. The debris adhering to the gland, such as the enteron and fat, was removed using a razor blade or forceps, and excess physiological saline was removed with a piece of filter paper. One drop of 5% methylene blue trihydrate was added to stain the gland for 1 minute, and a cover slip was placed on top of the specimen to make it ready for microscopic examination.

**Observations under the electron microscope.** The larvae were dissected as described above, and then prepared for observation as follows. The sialisterium was placed in 3% glutaraldehyde overnight at 4° C, and washed twice with 0.2 mol/L phosphate buffer (letting it remain in the buffer for 30 minutes each time). Then, it was fixed in 1% osmic acid for 2 hours at 4° C, washed with 0.2 mol/L phosphate buffer two or three times to remove the osmic acid, and dehydrated by passing it twice through a series of dilutions of acetone (50% for 10 minutes, 70% for 10 minutes, 90% for 10 minutes, and 100% for 15 minutes). The dehydrated specimens were left in a mixture comprising equal parts of acetone and Epon 812 epoxide resin for 30 minutes, and then kept overnight in Epon 812 epoxide resin. After putting the specimen in Epon 812 epoxide resin, it was polymerized for 36 hours at 60° C, and positioned for semithin sections in the sectioning machine. The specimens were sliced with a diamond cutter (LKBV ultramicrotome, LKB, Sweden). Each slice was placed on a copper screen, double-stained with 3% uranyl acetate for 60 minutes, and lead citrate for 20 minutes. Then, they were washed with deionized water, and dried at room temperature to make it ready for observation under the electron microscope. Organelles such as the nucleus, mitochondria, dictyosomes, ribosomes, and the endoplasmic reticulum were observed under the electron microscope.

## Results

### The morphology of silk glands in *S. mosellana* (Gehin)

**Figure 1.**
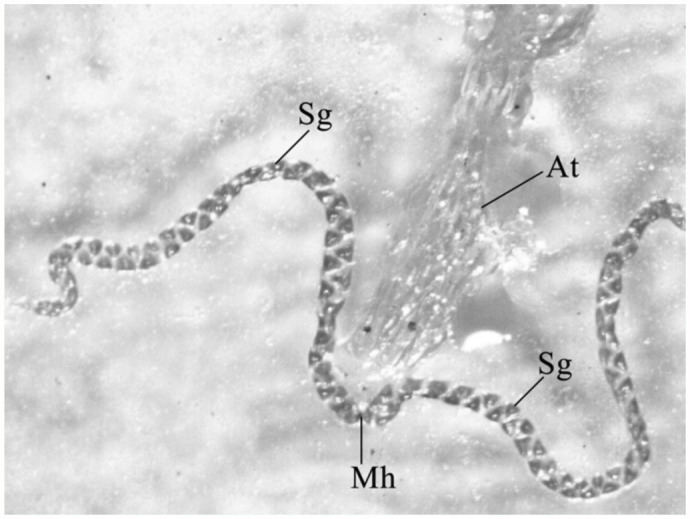
The morphology of of silk glands in *Sitodiplosis mosellana.* 600x. (Sg) Silk gland. (At) Alimentary tract. (Mh) Mouth hook. High quality figures are available online.

The silk gland of *S. mosellana* was hyaline, connected with the mouth hook, and comprised of two halves (left and right) at the sides of the alimentary tract. The cells of the gland were triangular, and granular in consistency, with transparent cell junction spread across the cell and interspersed with the granules. This arrangement made the gland perisome appear rippled, and the gland appear like a twisting lateral (Figure 1). Each gland consisted of 156 cells, with 78 cells on each side (n=30).

### Ultrastructure of the silk glands in *S. mosellana*

**Figure 2.**
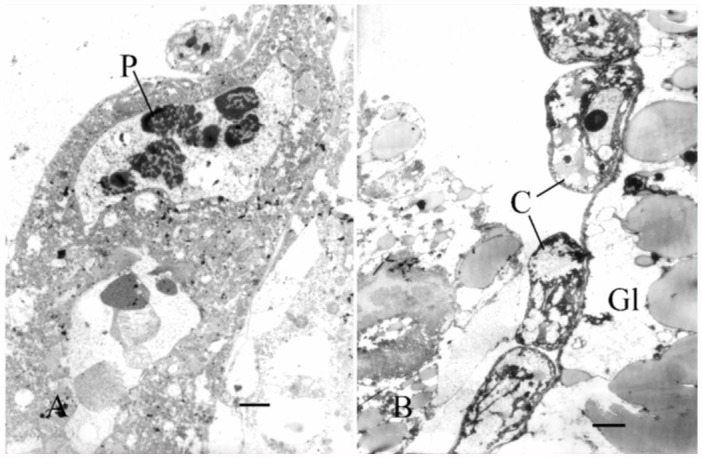
Silk glands cell of *Sitodiplosis mosellana.* A-Polykaryocytes(P). Bar = 1 mm. B- Gland cells had a compact location. (C) cells. (Gl). Gland lumen Bar = 5 mm. High quality figures are available online.

Each cell of the silk gland was a polykaryocyte (Figure 2A) with well-developed organelles, such as the endoplasmic reticulum, Golgi apparatus, mitochondria, and the silk gland lumen. The organelles were arranged compactly around the gland lumen in single file (Figure 2B). Such structure showed that the silk gland cell was particularly well adapted for secretion, and was closely related to the habit of cocooning for single, as well as multiple, diapause of the larva. However, at the same time, compared to the organelles in the gland cells in diapause larvae, organelles in the non-diapause larvae were more developed, and had a different structure, which proves that the vital movement of non-diapause larvae was in a more active condition.

### Endoplasmic reticulum

**Figure 3.**
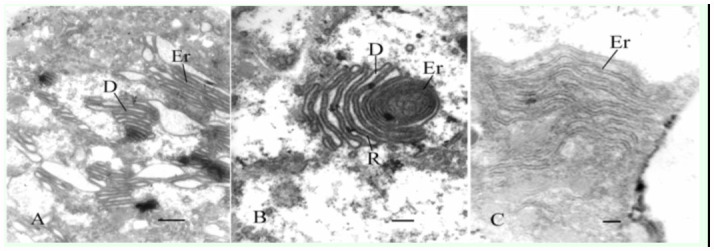
Endoplasmic reticulum in silk gland cell of *Sitodiplosis mosellana* larva. A- The complex endoplasmic reticulum of non-diapause larva. Bar = 500 nm. B- Ring-shaped endoplasmic reticulum of non-diapause larva. Bar = 200 nm. C- Endoplasmic reticulum of diapause larva. Bar = 200 nm. (Er). Endoplasmic reticulum. (D). Dictyosome. (R) Ribosomes. High quality figures are available online.

The endoplasmic reticulum in a non-diapause larva (Figures 3A and 3B) were more developed than in a diapause larva (Figure 3C), and the structures formed with the endoplasmic reticulum and the dictyosome in the non-diapause larva were more complex. The endoplasmic reticulum was electro-dense, and structured in a ring-shape, like a wheel (Figure 3B). Ribosomes were distributed copiously along the endoplasmic reticulum, geared for assembling proteins. The endoplasmic reticulum in the diapause larvae was discontinuous, structurally simple, and without ribosomes (Figure 3C).

### Dictyosome

**Figure 4.**
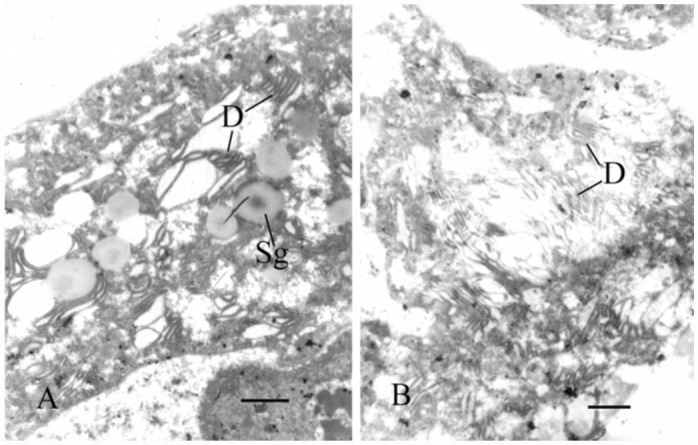
Dictyosome in silk gland cell of *Sitodiplosis mosellana* larva. A- non-diapause larva. Bar = 1 mm. B- diapause larva. Bar = 1 mm. (D). Dictyosome. (Sg) Secretory granule. High quality figures are available online.

Dictyosomes in the silk gland cells of non-diapause larva (Figure 4A) were electro-dense, with many large secretory granules. Furthermore, they were greater in number, and better developed than the dictyosomes in the diapause larvae (Figure 4B). The secretory granules in the non-diapause larva may have been protein granules, which can be processed and transported to the cytoplasm when the larva enters diapause. The protein granules can be transported from the cytoplasm to the gland lumen, and secreted as the silk cocoon is formed.

### Mitochondria

**Figure 5.**
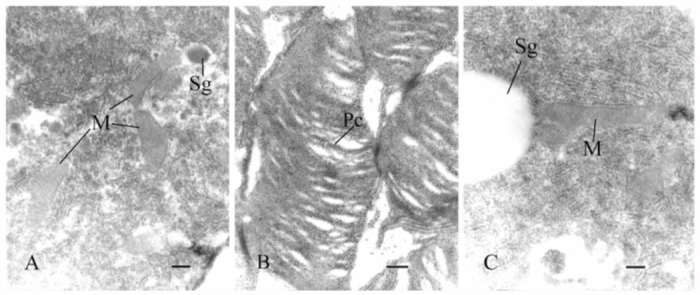
Mitochondria in silk gland cell of *Sitodiplosis mosellana* larva. A- Non-diapause larva. Bar = 200 nm. B- The enlarged drawing of mitochondria in silk gland cell of non-diapause larva. Bar = 100 nm. C- Diapause larva. Bar = 200 nm. (M) Mitochondria. (Sg) Secretory granule. (Pc) Parallel cristae. High quality figures are available online.

Silk gland cells of non-diapause larvae were rich in mitochondria. Around the mitochondria, there were some secretory granules, which were highly electro-dense (Figure 5A). The enlarged mitochondria were oblong, and several parallel cristae were seen at their centers (Figure 5B). Silk gland cells of diapause larvae contained only a few mitochondria, and the secretory granules around the mitochondria were vacuolated (Figure 5C). The mitochondrial structure of non-diapause larva were geared to provide abundant energy for the synthesis of fibroin.

### Lipid droplet and glycogenosome

**Figure 6.**
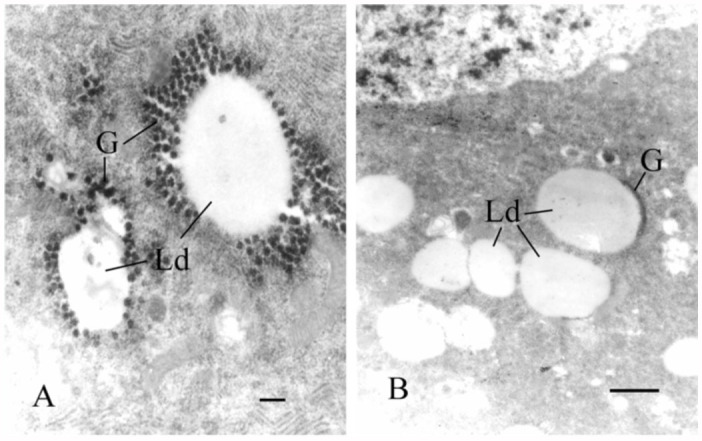
Lipid droplet and glycogenosome in silk gland cell of *Sitodiplosis mosellana* larva. A- Non-diapause larva. Bar = 200 nm. B-diapause larva. Bar = 500 nm. (Ld) Lipid droplet. (G) Glycogenosome. High quality figures are available online.

Lipid droplets and glycogenosomes were clearly visible in the silk gland cells of both diapause and non-diapause larvae. The droplets in non-diapause larvae were larger, but had a smaller quantity of lipid, and were surrounded by numerous glycogenosomes (Figure 6A). In the silk gland cells in diapause larvae, the droplets were smaller, but had a larger quantity of lipid, and glycogenosomes were either absent or very few (Figure 6B). Glycogen is a major, and readily convertible, source of stored energy. Cells that are rich in glycogen are ideal for supporting the synthesis of fibroin that occurs prior to cocooning, in the case of non-diapause larvae. The larger quantity of lipid in the droplets, in the case of diapause larvae, shows that the lipids were present as glycerol and fat, meant to ensure the insect's survival under adverse environmental conditions during the diapause.

**Figure 7.**
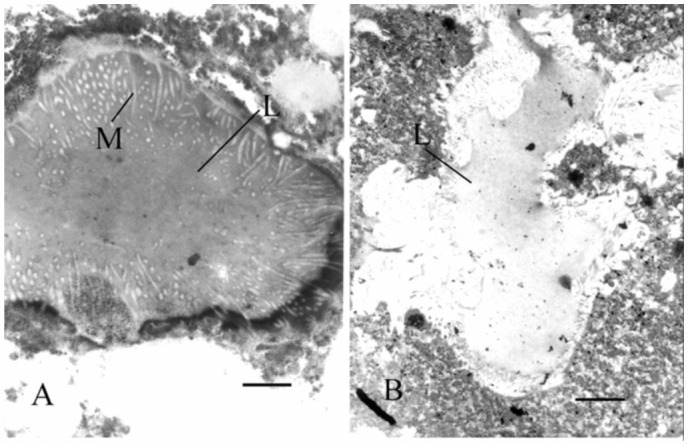
The material in the lumen of silk gland in silk gland cell of *Sitodiplosis mosellana* larva. A- Non-diapause larva. B- diapause larva. (L) Lumen. (M) Microvillus. Bar = 1 mm. High quality figures are available online.

**The material in the lumen of the silk gland** The silk gland in the non-diapause larvae had better-developed organelles in the cytoplasm than the diapause larvae, and it had a developed lumen (Figure 7A). The lumen of the silk gland in non-diapause larvae was filled with a silk-like, electro-dense substance, and featured a microvillus (Figure 7A). In diapause larvae, the lumen contained only small quantities of the substance, and the substance it did have was less electro-dense (Figure 7B). The rich, silk-like substance in non-diapause larvae can meet the demands of fusules and cocooning. In diapause larvae, this demand is absent.

## Discussion

The structure of insect silk glands has been well studied in Lepidoptera (Akai 1984), Hymenoptera (Silva-Zacarin 2003, Serrão 2005, Victoriano 2007), and especially *Bombyx mori*, because its silk has great economic value (Iijimaa 1972, Sasaki and Tashir 1976, Sehnal and Akai 1990). The above studies indicated that the major components of the silk or cocoon secreted by the silk gland were friboin and sericin (Akai 1984). Victoriano (2007) suggested that the silk produced by *Diatraea. saccharalis* has minor lipid contents and sericin, and that the lipid makes the cocoon waterproof. The fibroin, sericin, and lipid are synthesized respectively by endoplasmic reticulum, dictyosome and lipid droplets of the silk gland cell, and eventually they are secreted into the glandular lumen (Sasaki and Tashir 1976, Victoriano 2007). There is only one report of silk gland studies in Diptera (Young and Merrit 2003). Our study showed that the silk gland of *S. mosellana* was made up of 156 cells, 78 cells in each half. The organelles such as mitochondria, dictyosomes, ribosomes, and the endoplasmic reticulum were well developed, and the gland cells were organelle rich. The compound structure of dictyosomes and the endoplasmic reticulum is particularly well adapted for secretion of silk. Such characteristics are closely related to the habit of cocooning in diapause. Lipid droplets were clearly visible in the silk gland cells of *S. mosellana*, so we may conclude that the cocoon of diapause larvae may also have lipid components. This result was consistent with Victoriano's research that the silk gland can secrete lipid (Victoriano 2007).

Hu et al. (1988) found that the greater the frequency of cocooning in *S. mosellana*, the higher the consumption of the contents of the gland, resulting in a looser, granular structure, and gradually loosing the lumen. After cocooning three times, the granular structure was hardly visiblem and thus the contents in gland peristome decreased, the ripple-like appearance changed into isolated strands, flaccid, flat, and eventually atrophies. Zhong et al. (1980), and Dai (2001) reported that in *B. mori,* the organelles, such as the endoplasmic reticulum, in the cytoplasm of the silk gland cells degenerate after the larva spins the cocoon. Our results also showed that the silk gland cell in diapause larvae of *S. mosellana* had poorly developed organelles, which is in agreement with previous studies. The structure of silk gland cells are closely related to the frequency of cocooning and diapauses, which suggests that the silk gland plays an important role in *S. mosellana* diapause.

Compared to the organelles in silk gland cells of a diapause *S. mosellana* larvae, the organelles in a non-diapause larva were more developed, stimulated by the more active vital movement in the non-diapause larva. Our studies suggest that the larval behavior of different types of larvae could be accurately distinguished. This information will be useful for development of strategies to manage this important insect pest by determining the development stages of *S. mosellana* so as to forecast more accurately its population dynamics.
